# Overexpression of *IbFAD8* Enhances the Low-Temperature Storage Ability and Alpha-Linolenic Acid Content of Sweetpotato Tuberous Roots

**DOI:** 10.3389/fpls.2021.764100

**Published:** 2021-10-29

**Authors:** Chan-Ju Lee, So-Eun Kim, Sul-U Park, Ye-Hoon Lim, Chang Yoon Ji, Hyun Jo, Jeong-Dong Lee, Ung-Han Yoon, Ho Soo Kim, Sang-Soo Kwak

**Affiliations:** ^1^Plant Systems Engineering Research Center, Korea Research Institute of Bioscience and Biotechnology (KRIBB), Daejeon, South Korea; ^2^Department of Environmental Biotechnology, KRIBB School of Biotechnology, University of Science and Technology (UST), Daejeon, South Korea; ^3^R&D Center, Genolution Inc., Seoul, South Korea; ^4^Department of Applied Biosciences, Kyungpook National University, Daegu, South Korea

**Keywords:** abiotic stress, alpha-linolenic acid, *IbFAD8*, low temperature, sweetpotato, tuberous roots

## Abstract

Sweetpotato is an emerging food crop that ensures food and nutrition security in the face of climate change. Alpha-linoleic acid (ALA) is one of the key factors affecting plant stress tolerance and is also an essential nutrient in humans. In plants, fatty acid desaturase 8 (FAD8) synthesizes ALA from linoleic acid (LA). Previously, we identified the cold-induced *IbFAD8* gene from RNA-seq of sweetpotato tuberous roots stored at low-temperature. In this study, we investigated the effect of IbFAD8 on the low-temperature storage ability and ALA content of the tuberous roots of sweetpotato. Transgenic sweetpotato plants overexpressing *IbFAD8* (TF plants) exhibited increased cold and drought stress tolerance and enhanced heat stress susceptibility compared with non-transgenic (NT) plants. The ALA content of the tuberous roots of TF plants (0.19 g/100 g DW) was ca. 3.8-fold higher than that of NT plants (0.05 g/100 g DW), resulting in 8–9-fold increase in the ALA/LA ratio in TF plants. Furthermore, tuberous roots of TF plants showed better low-temperature storage ability compared with NT plants. These results indicate that *IbFAD8* is a valuable candidate gene for increasing the ALA content, environmental stress tolerance, and low-temperature storage ability of sweetpotato tuberous roots *via* molecular breeding.

## Introduction

The ongoing global climate change threatens crop productivity by affecting plant physiological processes and reducing plant biomass (Aryal et al., 2019). The availability of water, a major factor limiting agricultural production, is predicted to substantially decrease by 2050 (UN-Water, 2013). Approximately 15 billion tons of nutrient-rich soil is degraded each year because of climate change (Arora, 2019). According to the Food and Agriculture Organization (FAO), the global population will exceed 9.7 billion by 2050, which will increase the food demand by 1.7-fold if the current rate of food consumption is maintained (Food and Agriculture Organization [FAO], 2018). Therefore, to avoid food shortages in the future, the development of stress tolerant and high yielding crops is critical.

Sweetpotato (*Ipomoea batatas* [L.] Lam) is regarded as the fifth most important starch-rich crop that shows high potential for achieving the sustainable development goals, despite the ongoing climate change, owing to its high stress tolerance and ease of cultivation (Food and Agriculture Organization [FAO], 2020; Heider et al., 2021). Sweetpotato shows superior carbohydrate production capability than other starch-rich crops, such as wheat (*Triticum aestivum*), corn (*Zea mays*), potato (*Solanum tuberosum*), and cassava (*Manihot esculenta*), when grown on marginal lands (Ziska et al., 2009). Moreover, sweetpotato has been named as one of the top 10 superfoods important for human health, as it is a plentiful source of dietary fiber, potassium, and low-molecular-weight antioxidants such as carotenoids and vitamin C (CSPI, 2016). Thus, sweetpotato has emerged as an important food crop that ensures global food and nutrition security in the face of climate crisis (Kwak, 2019).

Given its tropical origin, sweetpotato is vulnerable to low temperature. East Asian countries account for approximately 70% of the global sweetpotato production, of which 65% is obtained from China. Because the sweetpotato crop is harvested in late autumn and early winter in East Asia, its tuberous roots are prone to low temperature stress during postharvest storage. The sweetpotato crop grown at high latitude over 120 frost-free days produces a higher yield of tuberous roots than that grown in tropical and subtropical regions. Exposure of sweetpotato tuberous roots to temperature below 10°C causes chilling injury, characterized by surface pitting, dry matter loss, lack of latex exudation, and increased susceptibility to decay, leading to yield losses. Therefore, the harvested sweetpotato tubers must be stored in a temperature-controlled storage facility. Expanding sweetpotato storable temperature will reduce greenhouse gas emissions and the maintenance cost occurred in the facility operation, thus enabling eco-friendly and economical postharvest storage of sweetpotato. Molecular profiling of sweetpotato has been recently conducted to understand its cold tolerance mechanism and to improve its low-temperature storage ability (Ji et al., 2017a, 2019, 2020).

Plants are sessile organisms and have therefore evolved unique mechanisms to survive various abiotic and biotic stresses. Plant cell membrane serves as a physical barrier against stress-induced damage by regulating membrane fluidity (Wang et al., 2006; Los et al., 2013). The degree of membrane fluidity is determined by the level of desaturated fatty acids, which is mainly regulated by the conversion between dienoic and trienoic acids. Expression levels of genes encoding desaturase enzymes also determine the content of desaturated fatty acids (Los et al., 1993). As representative ω-3 desaturase enzymes, microsomal endoplasmic reticulum (ER)-localized fatty acid desaturase 3 (FAD3) and plastidial FAD7 and FAD8 are responsible for the biosynthesis of alpha-linolenic acid (ALA; ω-3 fatty acid, C18:3) from linoleic acid (LA; ω-6 fatty acid, C18:2). These enzymes introduce additional double bonds to methyl end ω-3 (carboxylic end Δ15) positions in the long LA chains to synthesize ALA.

Having a high affinity to 18:2 acyl-lipid substrates rather than to 16:2 and a higher preference to phosphatidylglycerol (PG) than galactolipids, a membrane-bound FAD8 plays a crucial role in trienoic acid synthesis in leaves (Matsuda et al., 2005; Román et al., 2015). Transcript levels of *FAD8* are responsive to various environmental stresses; in many plant species, *FAD8* is upregulated by cold (Shi et al., 2011) and downregulated by heat and drought stress (Gopalakrishnan Nair et al., 2009; Xu et al., 2018). Moreover, changes in abiotic stress tolerance and trienoic acid levels have been confirmed in *FAD8* transgenic plants. For example, transgenic rice (*Oryza sativa*) deficient in *OsFAD8* is susceptible to cold stress, indicating that OsFAD8 plays an important role in low temperature acclimation (Tovuu et al., 2016). On the contrary, overexpression of the *Arabidopsis thaliana AtFAD8* gene in tobacco (*Nicotiana tabacum*) increased susceptibility to heat stress, but enhanced phenotypes under drought conditions (Zhang et al., 2005).

ALA, a representative ω-3 fatty acid, is essential for human health, as it is a precursor of important long-chain polyunsaturated fatty acids (LC-PUFAs) such as eicosapentaenoic (EPA; C20:5) and docosahexaenoic (DHA; C22:6). Because ALA has been shown to prevent cardiovascular disease, consumption of high amounts of ALA as well as EPA and DHA is recommended (Crawford et al., 2000). According to the National Academy Press (United States), adult males and females should consume 1.6 and 1.1 g ALA per day, respectively (Trumbo et al., 2002). Moreover, the general recommendation is to consume ω-6:ω-3 ratio as 5:1, however, the ratio in the modern diet is 15:1–20:1 (Simopoulos, 2001). Since ALA is not synthesized in the human body, it must be obtained from the diet. Although sea fish is rich in ω-3 LC-PUFAs, its supply is limited by heavy metal contamination (Liu et al., 2012). Oilseed crops such as flax, soybean, and walnut also contain abundant amounts of ω-3 LC-PUFAs. However, because the oxidation of PUFAs renders the oil product “unflavored,” the new oilseed plant genotypes developed contain low levels of ALA (Feussner and Wasternack, 2002; Vrinten et al., 2005; Clemente and Cahoon, 2009). Hence, alternative sources of ALA need to be identified.

Previously, we performed transcriptome profiling of sweetpotato tuberous roots during low temperature storage and excavated *IbFAD8*, one of several genes highly induced by cold (Ji et al., 2017a). In this study, we characterized the physiological functions of *IbFAD8*, as it might contribute to the modification of membrane lipids in tuberous roots during low temperature storage. We generated transgenic sweetpotato lines overexpressing *IbFAD8* (hereafter referred to as TF plants), and compared the ALA and LA contents of TF plants with those of non-transgenic (NT) plants. The ALA/LA ratio in the leaves of TF plants was slightly higher than that of NT plants, and was associated with enhanced cold and drought stress tolerance but reduced heat stress tolerance. Tuberous roots, the main edible part, showed higher ALA levels and ALA/LA ratio in TF plants than in NT plants. Moreover, tuberous roots of TF plants displayed enhanced low-temperature storage ability compared with those of NT plans. These results suggest that *IbFAD8* is a highly valuable gene that could be used for the molecular breeding of sweetpotato genotypes with high ALA contents that would be suitable for cultivation in high-latitude regions.

## Materials and Methods

### Plant Materials

Sweetpotato (*Ipomoea batatas* [L.] Lam. cv. Xushu 29) plants were grown in round pots filled with horticultural soil under controlled conditions (25°C temperature and 16-h light/8-h dark photoperiod). To regenerate transgenic lines, embryogenic calli were cultured in the dark at 25°C on Murashige and Skoog (MS) medium containing basal salt with 1 mg L^–1^ of 2, 4-dichlorophenoxyacetic acid (2, 4-D).

### Sequence Alignment and Phylogenetic Tree Analysis

Based on the deduced amino acid sequence of IbFAD8 (GenBank accession number: MZ287863), *FAD* family genes were identified by BLAST searches on the National Center for Biotechnology Information (NCBI) website. Then, amino acid sequences of IbFAD8, AtFAD7, AtFAD8, OsFAD7, and OsFAD8 were aligned using the BoxShade server.^1^ To clarify phylogenetic relation of IbFAD8 with that of other crops, a phylogenetic tree was constructed using the neighbor-joining method with 1,000 bootstrap replicates using Molecular Evolutionary Genetics Analysis version 7 (MEGA 7).

### Subcellular Localization of IbFAD8

The *IbFAD8* gene was cloned into the pGWB5 vector (Invitrogen, United States) containing the *GFP* gene. The *IbFAD8-GFP* construct was introduced into *Agrobacterium tumefaciens* strain GV3101, and the transformed cells were used to inoculate the leaves of 3-week-old tobacco plants, as described previously (Ji et al., 2016). Two days post-inoculation, the fluorescence signals of IbFAD8-GFP and chlorophyll a were detected in the abaxial surface of leaves using a Leica TCs SP2 confocal microscope (Leica Microsystems, Heidelberg, Germany).

### Gene Expression Analysis

Total RNA was extracted from frozen ground tissues of sweetpotato plants using the TRIzol Reagent (Invitrogen, MA, United States), according to the manufacturer’s instructions. Then, cDNA was synthesized from the isolated RNA using TOPscript^TM^ RT DryMIX (dT18) (Enzynomics, Daejeon, South Korea). To quantify gene expression levels, qRT-PCR was conducted on a Bio-Rad CFX Connect Real-Time PCR Detection System (Bio-Rad Laboratories, Hercules, CA, United States) using 2X Real-Time PCR Master Mix and EvaGreen^TM^ dye (BioFACT, Daejeon, South Korea). The conditions for the qRT-PCR was as follows: initial denaturation for 15 min at 95°C, followed by 45 cycles of 95°C for 20 s, 58°C for 40 s, and 72°C for 20 s. Relative gene expression were calculated using the comparative delta-delta Ct method, and the *Ubiquitin* gene was used as an internal control for the normalization of *IbFAD8* expression levels. The transcript levels were expressed as relative values, which the lowest expression was valued as 1. The sequences of primers used for qRT-PCR are listed in Supplementary Table 1.

### Generation of *IbFAD8* Overexpression Lines

To generate transgenic sweetpotato lines overexpressing *IbFAD8*, the coding sequence (CDS) of *IbFAD8* was cloned into the modified pCAMBIA1300 expression vector. The *IbFAD8* overexpression vector was transformed into the embryogenic callus of sweetpotato (cv. Xushu 29) *via Agrobacterium*-mediated transformation, as described previously (Lim et al., 2007). The transformed callus was cultured on MS medium containing vitamins, 400 mg L^–1^ cefotaxime, and 25 mg L^–1^ hygromycin. TF lines were identified by PCR amplification of gDNA isolated from the leaves of regenerated plants. Transcript levels of *IbFAD8* in various TF lines were examined by qRT-PCR.

### Abiotic Stress Treatments

To investigate the role of IbFAD8 in abiotic stress tolerance in sweetpotato, 5-week-old TF and NT plants were subjected to cold (4°C), heat (42°C), and drought stress for 70 h, 60 h, and 12 days, respectively. The experiments were conducted with 3 replicates with 6 sweetpotato plants in each repeat. Prior to cold and heat stress treatments, all plants were fully hydrated to avoid dehydration stress during the experiments. After the stress treatments, plants were rehydrated and allowed to recover at 25°C (Ji et al., 2017a; Jin et al., 2017). In each treatment, leaf samples were harvested, frozen in liquid nitrogen, and stored at −70°C until needed for further analysis.

### Quantification of Photosynthetic Activity and Chlorophyll Contents

The 3rd and 4th leaves from the top of plants were used for measuring the photosynthetic activity (Fv/Fm) and chlorophyll contents. Fv/Fm, which represents the maximum quantum efficiency of photosystem II (PS II) was measured using the Handy PEA fluorometer (Hansatech, King Lynn, United Kingdom), and chlorophyll contents were measured using the chlorophyll meter SPAD-502 (Konica Minolta, Tokyo, Japan) (Park et al., 2020). Nine leaves per replicate were measured for both analyses.

### Fatty Acid Profiling of Leaves and Tuberous Roots by Gas Chromatography Analysis

The composition of fatty acids (16:0, 18:0, 18:1, 18:2, and 18:3) in leaves and tuberous roots of sweetpotato were analyzed by GC, as described previously (Beuselinck et al., 2006). Leaves from 5-week-old plants and tuberous roots from 5-month-old 3 individual sweetpotato were used for the experiments. An Agilent series 7890A capillary gas chromatograph equipped with a flame ionization detector at 250°C (Agilent Technologies Inc., Wilmington, DE, United States) was used to profile the fatty-acid methyl esters in the extracted oil. The extracts were obtained from freeze-dried samples using a 1.5-mL mixture of chloroform, hexane, and methanol (8:5:2, v/v/v) overnight. Then, 100 μL of the derivatization solvent was mixed with 75 μL of a methylation reagent (0.25 M methanolic sodium methoxide: petroleum ether: ethyl ether [1:5:2, v/v/v]). Samples were diluted with hexane to a final volume of ca. 1 mL. Five fatty acids were separated on a DB-FFAP capillary column (30 m × 0.25 mm, 0.25 μm; Agilent Technologies Inc., Wilmington, DE, United States). Standard fatty acid mixtures (Fame #16, RESTEK) were used as references for calibration. For fatty acid quantification, glyceryl triundecanoate (5 mg/ml) was used as internal standard.

### Measurement of Malondialdehyde and H_2_O_2_ Contents

MDA, a product of lipid peroxidation, is a commonly used indicator of oxidative stress in cells. A modified method using thiobarbituric acid (TBA) and trichloroacetic acid (TCA) was used to measure the MDA content (Puckette et al., 2007). Extracts in 0.1% TCA buffer obtained from 50 mg FW of sweetpotato tissues were mixed with 0.5% TBA buffer, and the mixture was heated in boiling water. The absorbance of each extract was recorded at 532 nm and 600 nm, and MDA content was calculated with an extinction coefficient of 155 mM ^–1^ cm ^–1^.

The relative H_2_O_2_ content of tuberous roots was measured by detecting a ferric-xylenol complex (Gay et al., 1999). Briefly, 100 mg FW of sweetpotato samples was extracted with 50 mM potassium phosphate buffer (pH 6.8). Assay buffer 1 was prepared by mixing 25 mM FeSO_4_, 25 mM (NH_4_)_2_SO_4_, and 2.5 M H_2_SO_4_, and assay buffer 2 was prepared by mixing 125 μM xylenol orange and 100 mM sorbitol. A homogenized mixture of sample extract, assay buffer 1, and assay buffer 2 was incubated at room temperature for 30 min, and optical density was measured at 560 nm. Both experiments were conducted three times with 3 independent samples in each replicate.

### Peroxidase Activity Assay

POD activity was determined with guaiacol as a substrate, as described previously (Kwak et al., 1995). Briefly, 0.1 g FW of sweetpotato tissues was homogenized in 0.1 M potassium phosphate buffer (pH 6.0) on ice. After centrifugation, protein concentration in the extracts was measured using a spectropho which comprise the active sites of desaturase enzymes tometer, according to the Bradford assay (Bradford, 1976). To determine the specific POD activity, 0.46% guaiacol, 13 mM H_2_O_2_, and 5 μg of crude protein were mixed with distilled water. The absorbance of the sample was recorded at 470 nm every 20 s for 3 min. The measurement was performed three times with 3 independent tuberous roots.

### Evaluation of Low-Temperature Storage Capacity of Tuberous Roots

To explore the effect of *IbFAD8* overexpression on the storage ability of tuberous roots under low temperature conditions, the tuberous roots of NT and TF lines were rinsed with tap water. After drying at room temperature, the roots were stored at normal (13°C) or cold (4°C) temperature in the dark (Ji et al., 2017a). After 6 weeks of storage, the roots were chopped, frozen in liquid nitrogen, and stored at −70°C until needed for analysis. The experiments were performed three times with more than 6 individual tuberous roots harvested from 5-month-old sweetpotato for each replicate.

### Statistical Analysis

Data were presented as mean ± standard deviation (SD). Data were subjected to the analysis of variance (ANOVA), followed by Tukey’s honestly significant difference (HSD) test.

## Results

### Isolation and Sequence Analysis of *IbFAD8* cDNA

**FIGURE 1:**
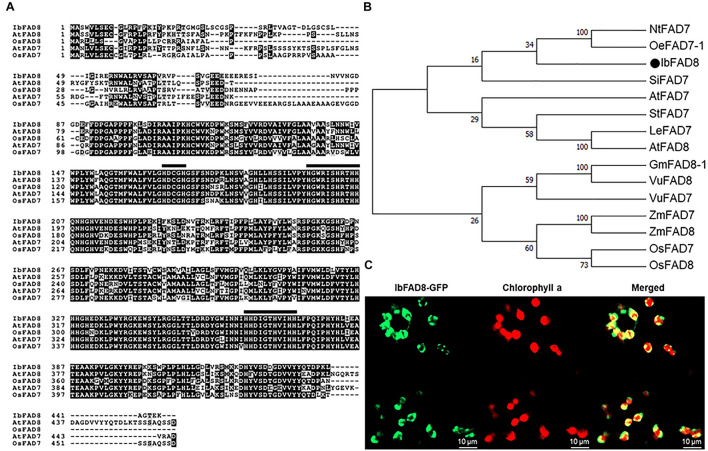
Amino acid sequence analysis of IbFAD8. **(A)** Alignment of the predicted amino acid sequences of FAD8 and FAD7 of various plant species. Ib, *Ipomoea batatas*; At, *Arabidopsis thaliana*, Os, *Oryza sativa*. **(B)** Phylogenetic analysis of the deduced amino acid sequences of plastidial FAD enzymes (FAD7 and FAD8) of various plant species. GenBank accession numbers of FAD sequences are as follows: NtFAD7 (BAC01274.1), OeFAD7-1 (AFX68461.1), SiFAD7 (NP_001306619.1), AtFAD7 (NP_187727.1), AtFAD8 (NP_196177.1), StFAD (NP_001274883.1), LeFAD7 (NP_001234592.1), GmFAD8-1 (NP_001238609.1), VuFAD8 (ABY60737.1), VuFAD7 (ABY60738.1), ZmFAD (BAA22441.1), ZmFAD8 (NP_001266540.1), OsFAD7 (BAE79783.1), OsFAD8 (AAW32557.1). **(C)** Subcellular localization of IbFAD8. The *IbFAD8-GFP* fusion was transiently expressed in *Nicotiana benthamiana* leaves. Confocal microscopy was used to visualize the GFP signal (green) and chlorophyll a autofluorescence (red). Merged images were used to confirm the subcellular localization of IbFAD8.

The *IbFAD8* cDNA was isolated from the sweetpotato cultivar Xushu 29 by homology-based BLAST searches against the transcriptome of cold-treated sweetpotato. The *IbFAD8* gene was predicted to encode a 445-amino acid (aa) protein. Amino acid sequence alignment of IbFAD8 with FAD7 (another plastidial ω-3 fatty acid desaturase) and FAD8 homologs of Arabidopsis and rice, as experimental models for dicots and monocots, respectively, showed three well-known conserved histidine clusters, HDCGH (166–170 aa), H—–HRTHH (196–206 aa), and HH—-HVIHH (363–373 aa) (Figure 1A), which comprise the active sites of membrane-bound desaturase enzymes (Wang et al., 2006). Moreover, phylogenetic analysis showed that IbFAD8 shares high sequence similarity with the plastidial ω-3 FADs, and is the most closely related to olive (*Olea europaea*) FAD7-1 (Figure 1B).

To determine the subcellular localization of IbFAD8, a translational fusion of *IbFAD8* with the *green fluorescent protein* (*GFP*) gene was transiently expressed in tobacco (*Nicotiana benthamiana*) leaves. Confocal microscopy showed that the IbFAD8-GFP signal (green) surrounded the chlorophyll a autofluorescence signal (red) (Figure 1C), indicating that IbFAD8 is a transmembrane protein in the chloroplast. Collectively, these results suggest that *IbFAD8* encodes a plastidial membrane-bound ω-3 fatty acid desaturase.

### Differential Expression of *IbFAD8* in Various Tissues and Under Different Stress Conditions

**FIGURE 2:**
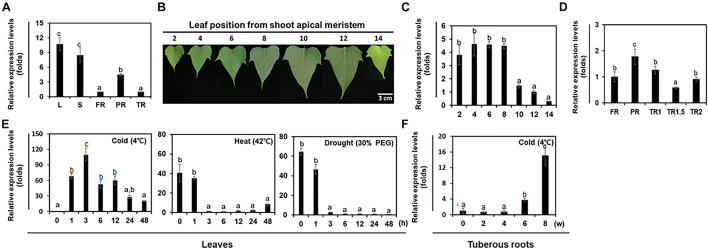
Analysis of the expression levels of *IbFAD8* in sweetpotato. **(A)** Expression levels of *IbFAD8* in different tissues of sweetpotato plants. The *Ubiquitin* gene was used as an internal control for the normalization of *IbFAD8* expression levels. L, leaves; S, stems; FR, fibrous roots; PR, pencil roots; TR, tuberous roots. **(B)** Position-dependent leaf development in sweetpotato plants. Images show representative leaves at each position from the shoot apical meristem. **(C)** Transcript levels of *IbFAD8* in leaves at different positions on the stem. **(D)** Expression levels of *IbFAD8* in sweetpotato tuberous roots at different developmental stages. FR, fibrous roots; PR, pencil roots; TR1, tuberous roots (diameter: 1–1.5 cm); TR1.5, tuberous roots (diameter: 1.5–2 cm); TR2, tuberous roots (diameter > 2 cm). **(E)** Transcript levels of *IbFAD8* in sweetpotato leaves and **(F)** tuberous roots in response to various abiotic stresses. The third and fourth leaves from the top of sweetpotato plants were treated with cold (4°C) and heat (42°C) stresses, and *IbFAD8* expression was analyzed by qRT-PCR. To analyze the effects of drought stress, the detached third leaf was placed in a 1.5-mL microfuge tube containing 30% polyethylene glycol (PEG 6000). Tuberous roots were stored at 4°C for 6 weeks to investigate *IbFAD8* expression levels. All the experiments were conducted three times with 3 individual tissues for each replicate. h, hours; w, weeks. Different lowercase letters indicate significant differences (*p* < 0.05; one-way ANOVA, followed by Tukey’s HSD *post hoc* test).

In various plant species, *FAD8* is expressed to higher levels in leaves than in stems and roots (Feng et al., 2017; Sun et al., 2018; Xue et al., 2018). In sweetpotato, we found that the expression level of *IbFAD8* was higher in aerial plant parts (leaves and stems) than in underground parts (fibrous, pencil, and tuberous roots) (Figure 2A). Among the various tissue types, leaves showed the highest *IbFAD8* transcript levels. We also investigated whether *IbFAD8* expression levels varied with leaf age (Figure 2B). The results showed that *IbFAD8* was expressed to similar levels in young (2nd and 4th) and mature (6th and 8th) leaves, but its expression level gradually decreased with increasing leaf age (10th, 12th, and 14th) (Figure 2C). In contrast to leaves, roots showed less development-dependent variation in *IbFAD8* expression (Figure 2D). These results suggest that *IbFAD8* expression shows a stronger correlation with leaf development than with root development in sweetpotato.

It has been shown that *FAD* gene expression is affected by physical and environmental stresses in various plant species, while simultaneously modifying the degree of desaturation of the cell membrane. In this study, we investigated the transcript levels of *IbFAD8* in sweetpotato tissues under abiotic stress conditions by quantitative real-time PCR (qRT-PCR) (Figure 2E). In leaves, the expression of *IbFAD8* was significantly induced by cold (4°C) stress, reaching a peak at 3 h, followed by a gradual decline. On the other hand, heat (42°C) and drought (30% polyethylene glycol [PEG]) stress treatments dramatically downregulated the transcript levels of *IbFAD8* in leaves, reaching the lowest level at 3 h. In tuberous roots, the expression of *IbFAD8* gradually increased during low temperature storage for 8 weeks (Figure 2F). These results suggest that *IbFAD8* transcript levels are affected by various environmental stresses and are significantly upregulated by cold stress.

### Generation of Transgenic Sweetpotato Lines Overexpressing *IbFAD8*

**FIGURE 3:**
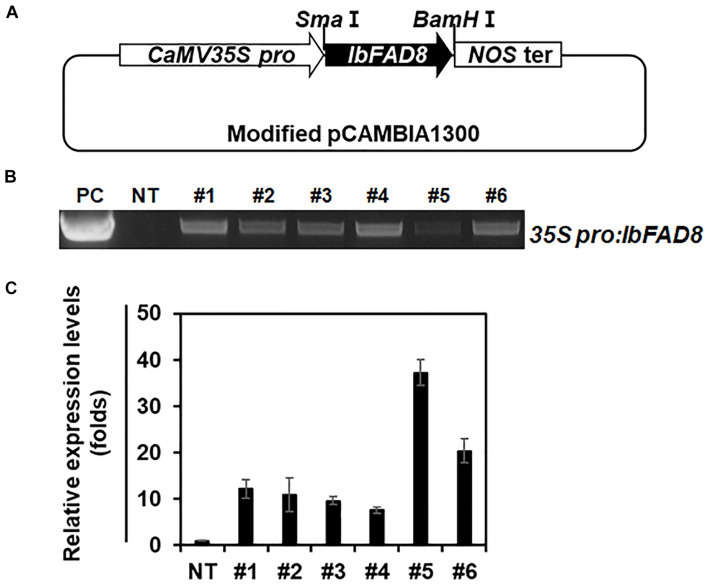
Generation of transgenic sweetpotato plants overexpressing *IbFAD8*. **(A)** Schematic diagram of the vector used to generate transgenic sweetpotato lines. The *IbFAD8* gene was expressed under the control of the cauliflower mosaic virus 35S (CaMV35S) promoter. **(B)** Gel image showing the results of genomic DNA (gDNA) PCR. PC, positive control; NT, non-transgenic plants. **(C)** Expression levels of *IbFAD8* in the leaves of transgenic lines. Lines #5 and #6 with high transcript levels (referred to as TF5 and TF6, respectively) were selected for further characterization.

To overexpress the *IbFAD8* gene in sweetpotato, a modified pCAMBIA1300 vector carrying the *IbFAD8* gene under the control of the cauliflower mosaic virus (CaMV) *35S* promoter was introduced into the embryogenic callus of the sweetpotato cultivar Xushu 29 *via Agrobacterium*-mediated transformation (Figure 3A). After selection on antibiotic-containing media, leaves generated from the transformed callus were subjected to genomic DNA (gDNA) PCR to identify transgenic lines (Figure 3B). Transgenic lines #5 and #6 (hereafter referred to as TF5 and TF6, respectively), with high transcript levels of *IbFAD8*, were selected for further characterization (Figure 3C).

### Increased Alpha-Linoleic Acid Contents of the Leaves and Tuberous Roots of TF Plants

Previously, changes in the expression level of *FAD8* in transgenic plants have been shown to modify the ALA and LA contents and consequently the ALA/LA ratio in leaves (Román et al., 2015; Tovuu et al., 2016). Here, we examined the fatty acid composition of the leaves of TF and NT plants by gas chromatography (GC) (Table 1). In NT leaves, ALA was the most abundant fatty acid (63.70%), followed by palmitic acid (15.83%), LA (15.01%), stearic acid (2.98%), and oleic acid (2.47%). In TF lines, the LA content was reduced by ca. 3–4%, whereas the ALA content was increased by ca. 1–2% compared with NT plants. Consequently, the ALA/LA ratio was ca. 1.2-fold higher in TF leaves than in NT leaves.

**TABLE 1:** Fatty acid composition of the leaves and tuberous roots of sweetpotato plants.

Tissue type	Genotypes^a^	Fatty acid composition^b^					ALA/LA ratio^b^
		Palmitic acid	Stearic acid	Oleic acid	Linoleic acid	Alpha-linolenic acid	
			
		(C16:0)	(C18:0)	(C18:1)	(LA; C18:2)	(ALA; C18:3)	
Leaves	NT	15.83 ± 0.35	2.98 ± 0.17	2.47 ± 0.02	15.01 ± 0.74	63.70 ± 0.24	4.25 ± 0.24
	TF5	16.93 ± 0.44**	2.99 ± 0.07	2.27 ± 0.18	11.65 ± 0.78**	66.14 ± 1.16	5.70 ± 0.47*
	TF6	17.51 ± 0.28**	2.92 ± 0.17	2.48 ± 0.26	12.56 ± 1.10*	64.53 ± 1.16	5.17 ± 0.57
Tuberous roots	NT	26.69 ± 0.85	4.62 ± 0.56	4.62 ± 0.56	53.39 ± 0.88	10.68 ± 0.18	0.20 ± 0.00
		(0.13 ± 0.02)	(0.02 ± 0.01)	(0.02 ± 0.01)	(0.27 ± 0.03)	(0.05 ± 0.01)	
	TF5	28.61 ± 3.10	4.75 ± 0.79	4.13 ± 0.31	24.75 ± 1.85**	37.76 ± 1.71**	1.53 ± 0.05**
		(0.14 ± 0.03)	(0.02 ± 0.01)	(0.02 ± 0.00)	(0.12 ± 0.00)	(0.18 ± 0.01)	
	TF6	26.69 ± 1.02	5.46 ± 1.06	4.07 ± 1.91	23.34 ± 2.02**	40.44 ± 1.23**	1.74 ± 0.14**
		(0.13 ± 0.01)	(0.03 ± 0.01)	(0.02 ± 0.01)	(0.11 ± 0.01)	(0.20 ± 0.01)	

*^*a*^NT, non-transgenic; TF5 and TF6; transgenic IbFAD8 overexpression lines #5 and #6, respectively.*

^*b*^Data are expressed as mole percentage for compositions based on peak areas obtained by gas chromatography (GC) analysis. Numbers in parentheses indicate content of each fatty acids (g/100 g). Values represent mean ± standard deviation (SD) of three independent replicates. *p < 0.05, **p < 0.01.

To explore the effects of *IbFAD8* overexpression on tuberous roots, TF plants were grown for 5 months in a growth chamber. The harvested roots were divided into two types: tuberous and pencil roots. No significant difference was observed in root development between NT and TF plants (Supplementary Figure 1). Being edible, tuberous roots were subjected to fatty acid profiling by GC analysis (Table 1). In the tuberous roots of NT plants, LA was the most abundant fatty acid (53.39%), followed by palmitic acid (26.69%) and ALA (10.68%). Interestingly, compared with NT lines, the ALA content of TF lines was approximately fourfold higher, while that of LA content was ca. twofold lower. Consequently, the ALA/LA ratio was ca. 8–9-fold higher in TF lines than in NT lines. Moreover, per 100 g tissue dry weight (DW), the amount of ALA was 3.8-fold higher in TF lines (0.19 g) than in NT lines (0.05 g).

### Abiotic Stress Tolerance of TF Plants

**FIGURE 4:**
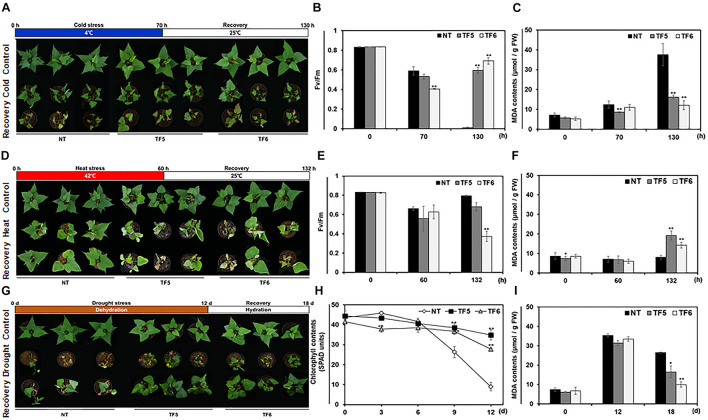
Evaluation of the abiotic stress tolerance of TF plants. **(A)** Phenotypic changes in TF plants exposed to cold (4°C) stress for 70 h, followed by recovery at 25°C for 60 h. **(B,C)** Fv/Fm values **(B)** and malondialdehyde (MDA) contents **(C)** of sweetpotato leaves after cold stress treatment and recovery. **(D)** Phenotypic analysis of TF plants treated with heat stress (42°C) for 60 h, followed by recovery for 72 h. **(E,F)** Fv/Fm values **(E)** and MDA contents **(F)** of sweetpotato leaves after heat stress treatment and recovery. **(G)** Phenotypes of TF plants treated with drought stress for 12 day, followed by rehydration for 6 day. **(H,I)** Chlorophyll contents (SPAD units) **(H)** and MDA contents **(I)** of sweetpotato leaves during drought stress treatment and recovery. Asterisks indicate significant differences between TF and NT plants (*p < 0.05, **p < 0.01).

It is well known that the modification of membrane desaturation levels due to changes in fatty acid composition and *FAD8* transcript levels affect tolerance to extreme temperatures in many plant species (Zhang et al., 2005; Wang et al., 2006). To evaluate the effect of *IbFAD8* overexpression on low temperature tolerance in sweetpotato, TF and NT plants were treated with cold (4°C) stress for 70 h, followed by recovery at a normal growth temperature (25°C) for 60 h, and the measurement of Fv/Fm values (Figures 4A–C). Although all plants were damaged by cold stress, TF plants showed slightly higher low-temperature tolerance than NT plants, especially in shoots (Supplementary Figure 2). After recovery, while the Fv/Fm values of NT plants could hardly be measured because of leaf necrosis, those of TF plants returned to normal levels (Figure 4B). Moreover, the level of malondialdehyde (MDA), a lipid peroxidation indicator, was 2.3–3-fold higher in NT plants than in TF plants after recovery (Figure 4C). These results indicate that TF plants exhibit greater cold stress tolerance than NT plants. Interestingly, opposite results were obtained in response to heat stress. After the 60-h heat (42°C) stress treatment, TF plants exhibited more susceptible phenotypes, characterized by leaf curling and severe leaf chlorosis, than NT plants (Figure 4D). Furthermore, the heat-treated TF plants showed higher MDA contents and lower Fv/Fm values than their NT counterparts; thus the TF plants showed little recovery, and some died at 25°C (Figures 4E,F). These results indicate that TF plants were more susceptible to heat stress than NT plants. Collectively, these data confirmed the contrasting responses of TF plants to cold and heat stress.

Previously, transgenic tobacco plants overexpressing *FAD8* were reported to show increased tolerance to drought tolerance, although the underlying mechanism remains unknown (Zhang et al., 2005). To evaluate the effect of *IbFAD8* overexpression on the drought tolerance of sweetpotato plants, NT and TF plants were dehydrated for 12 days (Figure 4G). The drought treated NT plants showed wilted and abscised leaves. Although the leaves of TF plants were also dehydrated after the drought stress treatment, they maintained their vitality, enabling the plants to fully recover following rehydration. Moreover, while the chlorophyll content of NT leaves decreased dramatically after the 6-day drought treatment, that of TF leaves showed a minimal decline (Figures 4H). Furthermore, it was confirmed that MDA contents of leaves were higher in NT plants than TF plants at recovery stage (Figure 4I). After rehydration, the leaves of TF plants were healthier and more turgid than those of NT plants, which retained only newly generated leaves. These results suggest that TF plants are more tolerant to drought stress than NT plants.

### Enhanced Low-Temperature Storage Ability of the Tuberous Roots of TF Plants

**FIGURE 5:**
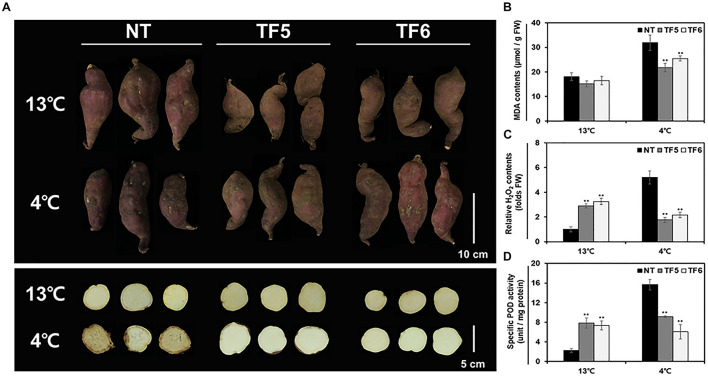
Evaluation of the low-temperature storage ability of the tuberous roots of sweetpotato plants. **(A)** Phenotypes of tuberous roots stored at normal temperature (13°C) and low temperature (4°C). Whole tuberous roots (upper panel) and root sections (lower panel) were photographed. **(B–D)** Quantification of MDA level **(B)**, hydrogen peroxide (H_2_O_2_) level **(C)**, and specific peroxidase (POD) activity **(D)** in tuberous roots after storage. Asterisks indicate significant differences between NT and TF plants (*p < 0.05, **p < 0.01; one-way ANOVA, followed by Tukey’s HSD *post hoc* test).

Because tuberous roots of sweetpotato are known to be susceptible to low temperature, many researchers have tried to understand the underlying molecular basis and to enhance the low temperature storage ability of tuberous roots (Ji et al., 2017a, 2020; Xie et al., 2019). In this study, tuberous roots of NT and TF lines were stored at 13°C (normal storage condition) and 4°C (low temperature condition) for 6 weeks, and their response to cold stress was examined. Only slight shrinkage and browning were observed on the surface of tuberous roots of NT and TF plants at 13°C, with no significant phenotypic differences between the two genotypes (Figure 5A). However, at 4°C, the tuberous roots of NT plants showed severe chilling injury symptoms and an increase in MDA and H_2_O_2_ contents (Figures 5B,C). On the contrary, tuberous roots of TF lines showed only slight phenotypic damage and lower MDA and H_2_O_2_ contents than those of NT plants. Interestingly, activity of the class III plant-specific oxidoreductase, peroxidase (POD), was threefold higher in TF lines than in NT lines at 13°C; however, at 4°C, POD activity was highly induced only in NT lines (Figure 5D). These results indicate low temperature storage ability of tuberous roots of TF was enhanced compared to NT tuberous roots.

## Discussion

Sweetpotato is valuable root crop ensuring world food security in the face of climate changes. However, sweetpotato, originated from tropical region, is susceptible to cold conditions during storage, which is an obstacle to sweetpotato industrialization. Therefore, it is urged to improve the storage ability of sweetpotato under low temperature. Moreover, whereas many researches have been performed to increase low-molecular-antioxidants, such as carotenoids, anthocyanins, and tocopherols in sweetpotato (Kim et al., 2020), there are lacks of researches on biofortification of alpha-linolenic acids (ALA), which are important nutrients for human health. Here, we investigated the functional roles of fatty acid desaturase 8 (FAD8) in terms of abiotic stress tolerance and fatty acid profiles in sweetpotato.

### Identification of *IbFAD8*

Plants possess two plastidial ω-3 fatty acid desaturases, FAD7 and FAD8, both of which are involved in ALA synthesis in the chloroplast envelope (Román et al., 2015). However, because of high sequence similarity between the two enzymes, their classification as FAD7 or FAD8 based on amino acid sequences is seldom easy. However, *FAD7* and *FAD8* exhibit differential responses to environmental stresses; while *FAD8* is induced by cold and repressed by heat stress, *FAD7* is downregulated or unresponsive to cold stress (Matsuda et al., 2005; Martz et al., 2006; Gopalakrishnan Nair et al., 2009; Román et al., 2015). Therefore, in previous studies, *FAD7* and *FAD8* have been distinguished based on their expression patterns in response to different stresses (Poghosyan et al., 1999; Hernández et al., 2016). In this study, the deduced protein sequence of IbFAD8 also showed three histidine clusters, associated with FAD7 and FAD8 enzyme activity, and high homology to FAD7 and FAD8 in other plant species (Figures 1A,B). Moreover, the deduced protein of IbFAD8 was localized in the chloroplast membrane (Figure 1C). Although domain analysis and subcellular localization results are not enough to determine whether the deduced protein is IbFAD7 or IbFAD8, we annotated the deduced amino acid sequence as IbFAD8 based on the cold-induced and heat-repressed expression of the isolated gene in leaves (Figures 1, 2E). As this study was focused on the low-temperature tolerance of tuberous roots of TF lines, we presented only cold-induced expressions of *IbFAD8* in tuberous roots under cold conditions. Transcript levels of *IbFAD8* in tuberous roots stored under high-temperature, including drought stress, will be investigated in further studies on the storage ability evaluation of TF line tuberous roots under heat and dry conditions.

### Overexpression of *IbFAD8* Altered the Abiotic Stress Tolerance of TF Plants

Fatty acid desaturation of membrane lipids under extreme temperature conditions is regulated by the expression levels of *FAD* genes to modify membrane fluidity (Los and Murata, 2004; Wang et al., 2006). Under low temperature, the degree of membrane fluidity is decreased, leading to the transition of membrane lipids from the liquid crystalline phase (normal) to gel-like phase (rigidified), which is accompanied by an increase in the ALA content and the upregulation of *FAD8* (Gibson et al., 1994; Los et al., 2013). On the other hand, high temperature increases the membrane fluidity and downregulates *FAD8* expression, thus increasing the LA content (Murakami et al., 2000; Román et al., 2015). The temperature stress-induced changes in *IbFAD8* expression observed in this study were in agreement with those observed in previous studies and might indirectly reflect the modification of membrane fluidity (Figure 2E). However, it was reported that fatty acid changes by FAD8 function under high-temperature conditions are attributed to post-transcriptional (Matsuda et al., 2005) or post-translational (Soria-García et al., 2019) mechanisms of FAD8 rather than its transcript levels. Therefore, for more detailed demonstrations, the expression analysis of IbFAD8 at the protein levels under diverse abiotic stresses should be performed, but it had been considered that there are barriers to studying FAD enzymes such as their high homology, high hydrophobicity, and restriction on employment of specific antibodies and purification methods. In a recent, those limitations were overcome by Soria-García et al. (2019) by using transgenic Arabidopsis expressing AtFAD7 and AtFAD8 fused to green fluorescent protein under the control of their endogenous promoters. Thus, transgenic sweetpotato producing IbFAD8-GFP fusion protein under the control of IbFAD8 promoter would be promising materials for the characterization of IbFAD8 protein expressions.

To investigate the function of *IbFAD8* in sweetpotato, transgenic sweetpotato lines overexpressing *IbFAD8* were generated (Figure 3). Leaves of TF plants exhibited a minor increase in the ALA/LA ratio (ca. 1%) compared with NT leaves (Table 1). Furthermore, TF plants showed slightly enhanced tolerance to cold stress but dramatically elevated vulnerability to heat stress (Figures 4A–F). It is well known that an increase in ALA content increases membrane fluidity, whereas increase in LA content promotes membrane rigidity (Berestovoy et al., 2020). Since fatty acid unsaturation has a major impact on temperature tolerance through the modulation of membrane fluidity (Zhang et al., 2005), we speculate that increased fatty acid unsaturation in TF plants might alleviate extreme rigidification under cold stress and, conversely, increase disorder with excessive fluidization under heat stress.

It has been suggested that membrane fluidity is decreased by hyperosmotic stress, similar to cold stress (Los and Murata, 2004). However, the correlation between drought stress tolerance and fatty acid composition (e.g., LA and/or ALA content or ALA/LA ratio) remains unclear. Zhang et al. (2005) suggested that drought stress-induced reduction in ALA content reflects plant damage, and increase in ALA content indicates plant defense. Based on this report, we expected that the downregulation of *IbFAD8* in sweetpotato leaves treated with 30% PEG might reflect the reduction in ALA content, indirectly leading to leaf tissue damage (Figure 2E). Moreover, the marked improvement in the drought tolerance of TF plants is presumably caused by the increased ALA/LA ratio in leaves (Figures 4G–I and Table 1), consistent with the *AtFAD8* overexpression-induced increase in the ALA/LA ratio of drought-tolerant transgenic tobacco plants (Zhang et al., 2005). In conclusion, the overexpression of *IbFAD8* in sweetpotato plants regulates abiotic stress tolerance by increasing the ALA content.

### Increased Accumulation of Alpha-Linoleic Acid in the Tuberous Roots of TF Lines

Previously, most studies focused on plant leaves to understand the physiological functions of FAD8 (Zhang et al., 2005; Wang et al., 2006; Gopalakrishnan Nair et al., 2009; Román et al., 2015; Tovuu et al., 2016). Therefore, information on the effect of *FAD8* on plant root physiology is limited. Consistent with previous studies (Abu et al., 2000; Wakita et al., 2001; Soto et al., 2014; Shen et al., 2018), we showed that the ALA/LA ratio was 0.2 in the tuberous roots of NT sweetpotato plants, and LA was the most abundant fatty acid (Table 1). However, ALA was the most abundant fatty acid in the tuberous roots of TF lines, and the ALA/LA ratio was 1.6, which is ca. 8-fold higher than the ALA/LA ratio in NT tuberous roots (Table 1). Additionally, the TF lines showed a 2.3-fold decrease and 3.8-fold increase in LA (0.12 g/100 g DW) and ALA (0.19 g/100 g DW) contents, respectively, compared with NT plants (cv. Xushu29) (Table 1); similar results were reported previously with the sweetpotato cultivar Nylon) (Soto et al., 2014).

ALA is an essential nutrient for human health, as it has been shown to prevent cardiovascular disease and promote neurological development and brain cell growth (Crawford et al., 2000). ALA also acts as a precursor of DHA and EPA. Humans can degrade ALA into DHA and EPA but cannot synthesize ALA; therefore, they must obtain ALA from external sources such as plants and sea fish. Considering that raw sweetpotato consists of 70% water, the dietary intake of one raw TF tuberous root [ca. 300 g fresh weight (FW)] supplies 12–18% of the recommended daily dose of ALA for adults (1.6 g/day for males, 1.1 g/day for females) (Trumbo et al., 2002). Shen et al. (2018) proposed the fermentation of sweetpotato with *Lactobacillus acidophilus* as a new biotechnological method for biofortification. This process promotes the accumulation of various nutrients, including ALA, in the extracts, which is effective in suppressing the proliferation of cancer cells without causing cytotoxicity to normal healthy cells. Therefore, fermentation of the tuberous roots of TF lines will increase their ALA contents compared with the raw tuberous roots of TF lines and NT plants. Collectively, these results suggest that the tuberous roots of TF lines represent a valuable and novel type of ALA-biofortified sweetpotato beneficial for human health.

### Enhanced Low-Temperature Storage Ability of the Tuberous Roots of TF Lines

The correlation between the ALA content of leaves and low temperature tolerance of plants has been well established to date. However, limited research has been conducted on the ALA content and low temperature tolerance of plant roots. To the best of our knowledge, this is the first report showing that sweetpotato tuberous roots with high ALA contents exhibit improved low-temperature storage ability (Figure 5). TF lines showed minimal phenotypic damage such as slight tissue browning, shrinkage, and decay after 6-week of storage at 4°C, which is consistent with the phenotype of cold tolerant sweetpotato (Ji et al., 2017b). We speculate that the enhanced cold tolerance of the tuberous roots of TF lines is caused by the increase in membrane fluidity in roots cells, which is attributed to ALA accumulation. Higher ALA levels in TF lines possibly increase membrane fluidity by alleviating excessive rigidification induced by cold stress.

Oxidative stress damages plant cells when the amount of reactive oxygen species (ROS) generated is higher than that scavenged by antioxidants (Zulfugarov et al., 2011). MDA and H_2_O_2_ contents, which are oxidative stress indicators, were lower in TF lines at 4°C compared with NT lines at 4°C (Figures 5B,C). The high level of H_2_O_2_ in TF lines at 13°C indicates that high amount of H_2_O_2_ is required as a substrate for POD, a multifunctional enzyme involved in oxidative stress tolerance *via* scavenging ROS (Kim et al., 2007, 2008; Li et al., 2011). TF lines showed higher POD activity than that of NT at 13°C (normal storage temperature), indicating enhanced basal oxidative stress tolerance (Figure 5D). These results suggest that the cells of TF lines coped well with ROS generation by chilling injuries under low temperature. On the other hand, NT plants exhibiting relatively lower POD activity at 13°C were hard to manage excessive ROS production at 4°C, resulting in severe chilling injury. Furthermore, stable POD activity in TF lines at 13 and 4°C reflects the physiological features of stress tolerant plants, contrary to NT plants, which showed a dramatic increase in POD activity at 4°C (Safizadeh et al., 2007; Huang et al., 2016). Consequently, we expected that, along with the increase in the content of the unsaturated fatty acid ALA, higher class III POD activity in TF lines might also contribute to their improved tolerance to low temperature *via* ROS scavenging. However, the reason for the increase in basal POD activity in TF lines remains unclear.

ALA in plants are abundant in the membrane of organelles involved in ROS production, such as chloroplast and mitochondria. Mene-Saffrane et al. (2009) reported that because of their sensitivity to ROS oxidation, trienoic acids participate in non-enzymatic ROS removal *via* the production of oxygenated molecules such as MDA. Moreover, the Arabidopsis *fad3*/*fad7*/*fad8* mutant shows greater vulnerability to fungal infection because of the lack of trienoic acids, suggesting that trienoic acids are involved in defense against pathogens (Mene-Saffrane et al., 2009). Therefore, it is possible that high ALA content of TF lines enhances low temperature tolerance by non-enzymatic ROS scavenging and pathogen attack as a secondary chilling injury in sweetpotato.

### Challenges in the Utilization of *IbFAD8* in Molecular Breeding Projects

FAD8, a chloroplast-localized enzyme, shows high affinity for phosphatidylglycerol (PG), which is the only phospholipid detected in leaf thylakoid and to which ALA is esterified at the sn-1 position (Román et al., 2015). Moreover, expression levels of plastidial ω-3 *FAD* genes are associated with photosynthetic green tissues, and the encoded enzymes catalyze the desaturation of LA in the chloroplast (Nishiuchi and Iba, 1998; Makarenko et al., 2011). However, several reports indicate that the ER-localized FAD3 is more functional in non-photosynthetic tissues than FAD8. In flax seeds, FAD3 is mainly involved in ALA biosynthesis (Vrinten et al., 2005). Wang et al. (2010) showed that FAD3 is the primary enzyme that synthesizes ALA in non-photosynthetic tissues in higher plants. A study showed that changes in the fatty acid profile of transgenic tobacco roots were more predominantly affected by the overexpression of *Brassica napus FAD3* (*BnFAD3*) than *AtFAD8* (Zhang et al., 2005). Given the predominant expression of *IbFAD8* in sweetpotato leaves, together with the absence of chloroplasts and an abundance of amyloplasts in plant roots, it is likely that IbFAD8 functions more specifically in leaves than in other tissues.

The results of this study suggest that overexpression of *IbFAD8* in sweetpotato causes more significant changes in tuberous roots than in leaves, with respect to the level of ALA accumulation and low temperature storage ability. We speculate that the dramatic physiological changes in the tuberous roots of TF lines resulted from the overexpression of the *IbFAD8*, which is otherwise expressed to low levels in non-photosynthetic tissues. To understand the mechanism of IbFAD8 function in further detail, it is important to determine the subcellular localization of IbFAD8 in root tissues. Furthermore, which of the three classes of lipids, neutral lipids, glycolipids, or phospholipids, are more relevant to the biosynthesis of ALA by IbFAD8 in root tissues needs to be elucidated in sweetpotato.

Since the TF lines showed enhanced tolerance to low temperature and dehydration, these might enable the expansion of sweetpotato cultivation into regions at high latitude and with harsh environments. Furthermore, *IbFAD8* overexpression did not have any negative impact on root development (Supplementary Figure 1). Additionally, tuberous roots of TF lines showed high ALA content and enhanced low temperature storage; these traits are beneficial for human health and the eco-friendly industrialization of sweetpotato production, respectively. The harvested sweetpotato tuberous roots are subjected to curing, an essential process that heals mechanical injuries and prevents pathogen attacks by storing the tuberous roots at 30–33°C temperature and 90–95% relative humidity for 3–5 days. However, because TF plants showed relatively high vulnerability to heat stress, it is possible that tuberous roots are also susceptible to high temperature because of the high ALA content. Therefore, further experiments should be performed in terms of curing process for successful industrialization of sweetpotato.

In conclusion, our results suggest that *IbFAD8* is a valuable gene that could be used to increase the ALA content and low-temperature storage ability of the tuberous roots of sweetpotato *via* molecular breeding approaches.

## Data Availability Statement

The original contributions presented in the study are included in the article/Supplementary Material, further inquiries can be directed to the corresponding author/s.

## Author Contributions

S-SK and C-JL conceived and designed the experiments. C-JL, S-EK, HJ, HK, S-UP, and Y-HL conducted the experiments. C-JL, S-EK, HK, U-HY, CJ, and J-DL analyzed the data. S-SK and J-DL provided the reagents, materials, and analysis tools. C-JL, S-EK, HK, and S-SK wrote the manuscript. All authors contributed to the article and approved the submitted version.

## Conflict of Interest

CJ was employed by the company Genolution. The remaining authors declare that the research was conducted in the absence of any commercial or financial relationships that could be construed as a potential conflict of interest.

## Publisher’s Note

All claims expressed in this article are solely those of the authors and do not necessarily represent those of their affiliated organizations, or those of the publisher, the editors and the reviewers. Any product that may be evaluated in this article, or claim that may be made by its manufacturer, is not guaranteed or endorsed by the publisher.

## Footnotes

1
https://embnet.vital-it.ch/software/BOX_form.html
