# Supplementary material for: Overexpression of IbFAD8 Enhances the Low-Temperature Storage Ability and Alpha-Linolenic Acid Content of Sweetpotato Tuberous Roots
Source: Front Plant Sci. 2021 Oct 29;12:764100. doi: 10.3389/fpls.2021.764100 (PMC8589035; doi:10.3389/fpls.2021.764100)
Supplement: Supplementary file 1 [file Data_Sheet_1.docx]

***Supplementary Material***

**Overexpression of *IbFAD8* enhances the low-temperature storage ability and alpha-linolenic acid content of sweetpotato tuberous roots**

***Chan-Ju Lee^1,2^, So-Eun Kim^1,2^, Sul-U Park^1,2^, Ye-Hoon Lim^1,2^, Chang Yoon Ji^3^, Hyun Jo^4^, Jeong-Dong Lee^4^, Ung-Han Yoon^1^, Ho Soo Kim^1*^, Sang-Soo Kwak^1,2*^***

^1^Plant Systems Engineering Research Center, Korea Research Institute of Bioscience and Biotechnology (KRIBB), Daejeon, Republic of Korea

^2^Department of Environmental Biotechnology, KRIBB School of Biotechnology, University of Science and Technology (UST), Daejeon, Republic of Korea

^3^R&D Center, Genolution Inc., Seoul, Republic of Korea

^4^Department of Applied Biosciences, Kyungpook National University, Daegu, Republic of Korea

*** Correspondence:**Dr. Sang-Soo Kwak
sskwak@kribb.re.kr

Dr. Ho Soo Kim
hskim@kribb.re.kr

**1. Supplementary Figures and Tables**

**1.1 Supplementary Figures**


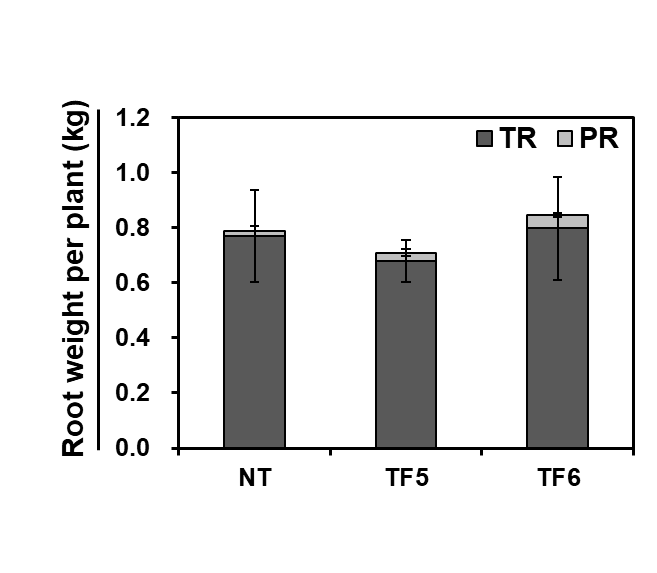


**Supplementary Figure 1.** Root weight (per plant) of 5-month-old sweetpotato plants. TR, tuberous roots; PR, pencil roots.


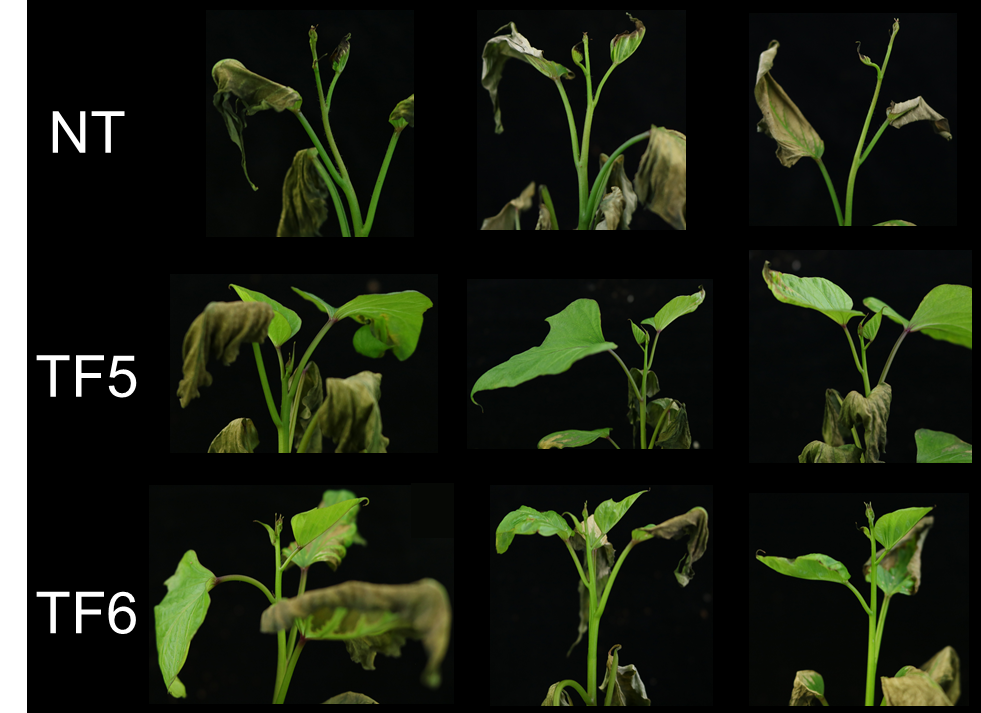


**Supplementary Figure 2.** Photographs of the shoots of cold-treated sweetpotato plants during recovery.

**2.2 Supplementary Table**

**Table S1.** List of gene-specific primers used in this study.

| Primer name | Primer sequence (5'→3') | Application |
| --- | --- | --- |
| IbFAD8_F | ATGGCGAGTTGGGTGTTATC | Vector construction |
| IbFAD8_R | TCACTTCTCGGTTCCAGCAA | Vector construction & genomic DNA (gDNA) PCR |
| pCAM1300_F | AGTGAGCGCAACGCAATTAATGTG | gDNA PCR |
| IbFAD8_F | CCCCCATTTAAGCTGTCTGA | Quantitative real-time PCR (qRT-PCR) |
| IbFAD8_R | ACATGGTTCCTTGAGCCAAC | qRT-PCR |
| Ubiquitin_F | TCGACAATGTGAAGGCAAAG | qRT-PCR |
| Ubiquitin_R | CTTGATCTTCTTCGGCTTGG | qRT-PCR |
